# Trifluoromethyl Boron Dipyrromethene Derivatives as Potential Photosensitizers for Photodynamic Therapy

**DOI:** 10.3390/molecules23020458

**Published:** 2018-02-19

**Authors:** Jian-Yong Liu, Peng-Zhen Zhou, Jia-Lin Ma, Xiao Jia

**Affiliations:** State Key Laboratory of Photocatalysis on Energy and Environment & National & Local Joint Biomedical Engineering Research Center on Photodynamic Technologies, College of Chemistry, Fuzhou University, Fuzhou 350108, China; 18714968018@163.com (P.-Z.Z.); MWY4512938@163.com (J.-L.M.); jiaxiao@fzu.edu.cn (X.J.)

**Keywords:** photosensitizer, BODIPY, photodynamic therapy, reactive oxygen species

## Abstract

In this study, two novel boron dipyrromethene-based photosensitizers (**BDP3** and **BDP6**) substituted with three or six trifluoromethyl groups have been synthesized and characterized with various spectroscopic methods, and their photo-physical, photo-chemical, and photo-biological properties have also been explored. The two photosensitizers are highly soluble and remain nonaggregated in *N*,*N*-dimethylformamide as shown by the intense and sharp Q-band absorption. Under red light irradiation (λ = 660 nm, 1.5 J/cm^2^), both photosensitizers show high and comparable cytotoxicity towards HepG2 human hepatocarcinoma and HeLa human cervical carcinoma cells with IC_50_ values of 0.42–0.49 μM. The high photocytotoxicity of **BDP3** and **BDP6** can be due to their high cellular uptake and low aggregation tendency in biological media, which result in a high efficiency to generate reactive oxygen species inside the cells. Confocal laser fluorescence microscopic studies indicate that they have superior selective affinities to the mitochondria and lysosomes of HepG2 and HeLa cells. The results show that these two trifluoromethyl boron dipyrromethene derivatives are potential anticancer agents for photodynamic therapy.

## 1. Introduction

Photodynamic therapy (PDT) is a clinically approved and non-invasive therapeutic procedure and has been entering the mainstream of cancer treatments [1,2,3,4,5,6]. PDT requires a photosensitizer (PS), light, and oxygen to initiate photo-chemical reactions that may result in the primary tumor destruction and provide protection against metastasis via the local generation of reactive oxygen species (ROS) [7,8,9]. An ideal photosensitizer with a high therapeutic efficacy should have the following suitable physicochemical properties: intense absorption in the phototherapeutic window (630–850 nm) [10], stable chemical structure and simple synthetic route, high ROS quantum yield, low dark toxicity and skin phototoxicity, easy clearance from the body, high selectivity to tumor cells, reasonable fluoro-/chemi-luminescence for effective tumor diagnostics, and favorable solubility in body’s fluids and injectable solvents [11,12,13]. In addition, the pharmacokinetics is also important for a new photosensitizer. The ideal photosensitizer should stably retain in the circulation for a sufficient time so that it can either localize in malignant tissues and/or their vasculature. Nowadays, the photosensitizers used in clinical practice are mainly tetrapyrrole macrocycles such as porphyrin [1,14], chlorin [15], and phthalocyanine [16,17]. However, the drawbacks of this class of molecules are the cumbersome synthesis and purification processes [18,19]. 

As versatile fluorescent dyes, boron dipyrromethenes (BODIPYs) are characterized by strong absorption in the visible and near-infrared region [9,20], high photo-stability, low sensitivity to environmental variation, and the easily modified basic core with tunable optical properties [21]. Heavy atoms (I [22,23], Br [24,25], Pt [26]) are incorporated into the BODIPY core to facilitate intersystem crossing, which can enhance the generation efficiency of singlet oxygen, and obtain a new candidate for photodynamic sensitizers [27,28,29]. Drug candidates with one or more fluorine atoms have become commonplace [10,30]. The incorporation of fluorine or trifluoromethyl groups into a drug allows the simultaneous modulation of electronic, lipophilic, and steric parameters. The lipophilicity of fluorine can enhance the cell membrane permeability so as to improve the bioavailability of drug candidates [31]. The strong electronegativity of fluorine can increase the binding force between the ligand and target, and optimize the pharmacodynamic properties of drugs. In addition, the stable C–F bond contributes to the metabolic stability of the drugs in body, therefore improving the pharmacokinetic properties of drugs [32,33]. However, incorporating fluorine or trifluoromethyl groups into the BODIPY core with a view to improving their therapeutic outcome has been seldom reported [34]. Herein, we demonstrate two novel BODIPY derivatives bearing 3 and 6 trifluoromethyl groups and named them **BDP3** and **BDP6**, respectively. Their photo-physical, photo-chemical, and photo-biological properties have also been explored. **BDP3** and **BDP6** show slight aggregation in the Roswell Park Memorial Institute (RPMI) 1640 cell culture medium, mainly exist in monomer form. Methyl thiazolyl tetrazolium (MTT) assays show that the two photosensitizers have the high photocytotoxicity against HepG2 and HeLa cancer cells with IC_50_ values of 0.42–0.49 μM. Subcellular localization experiments show that the two compounds are not found in the nucleus, but largely distribute in the mitochondria and lysosomes of HepG2 and HeLa cells. The two compounds with a different number of trifluoromethyl groups show almost the same photocytotoxicity. However, when we evaluate the performance of a new drug, cytotoxicity is not the only index. Its pharmacokinetics is also very important. **BDP6** containing more trifluoromethyl groups does not show a higher photocytotoxicity than **BDP3.** Maybe it has better pharmacokinetic characteristics. In the future, we will further explore the pharmacokinetic properties of the two BODIPY derivatives as suitable photosensitizers.

## 2. Results and Discussion

### 2.1. Molecular Design and Synthesis

**Scheme 1:**
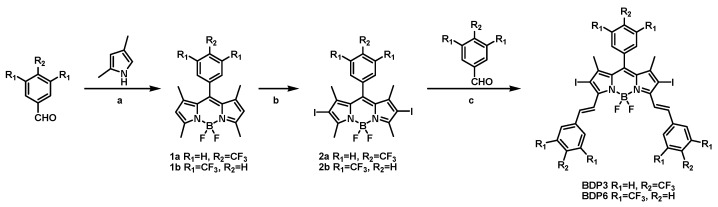
Synthetic route of **BDP3** and **BDP6**. a: (1) TFA, CH_2_Cl_2_ (2) 2,3-dichloro-5,6-dicyano-*p*-benzoquinone (3) Et_3_N, BF_3_·Et_2_O. b: I_2_, HIO_4_. c: piperidine, acetic acid, Mg(ClO_4_)_2_.

The syntheses of BODIPY derivatives **BDP3** and **BDP6** were accomplished by a multi-step synthetic procedure as shown in Scheme 1. The BODIPY mother compound **1a** or **1b** was first prepared by treating 2,4-dimethylpyrrole with 4-trifluoromethylbenzaldehyde or 3,5-bis(trifluoromethyl)benzaldehyde via sequential condensation, oxidation, and complexation reactions according to a classic procedure [21]. Both **1a** and **1b** were then treated with I_2_ and HIO_4_ in absolute ethanol to afford iodo-BODIPY **2a** and **2b**, respectively. Iodine atom is incorporated into the 2 or 6 position of the BODIPY mother core, which can facilitate intersystem crossing, therefore promoting the generation of singlet oxygen. Finally, compound **2a** or **2b** underwent a condensation reaction with 4-trifluoromethylbenzaldehyde or 3,5-bis(trifluoromethyl)benzaldehyde in the presence of piperidine and glacial acetic acid in toluene to give **BDP3** or **BDP6** in a moderate yield. Both compounds have a larger π-conjugated structure than their mother BODIPYs, showing strong absorbances in the red visible region, which is a desirable characteristic for efficient photosensitizers. All these new compounds were characterized by various spectroscopic methods.

### 2.2. Photo-Physical and Photo-Chemical Properties

**Figure 1:**
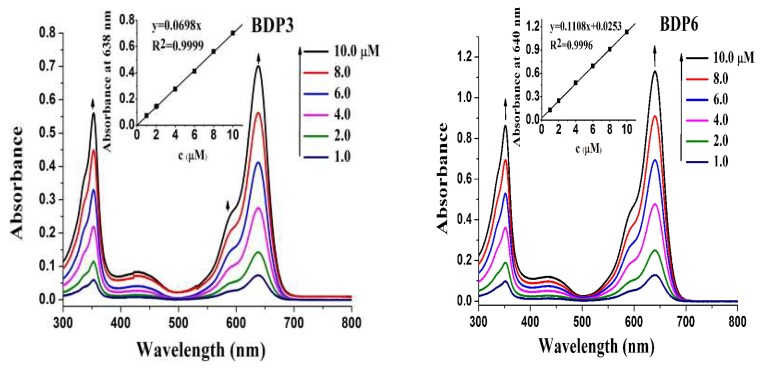
Electronic absorption spectra of **BDP3** and **BDP6** at various concentrations in DMF. The inset shows the variation of the Q-band absorbance with the concentration of BODIPYs.

**Figure 2:**
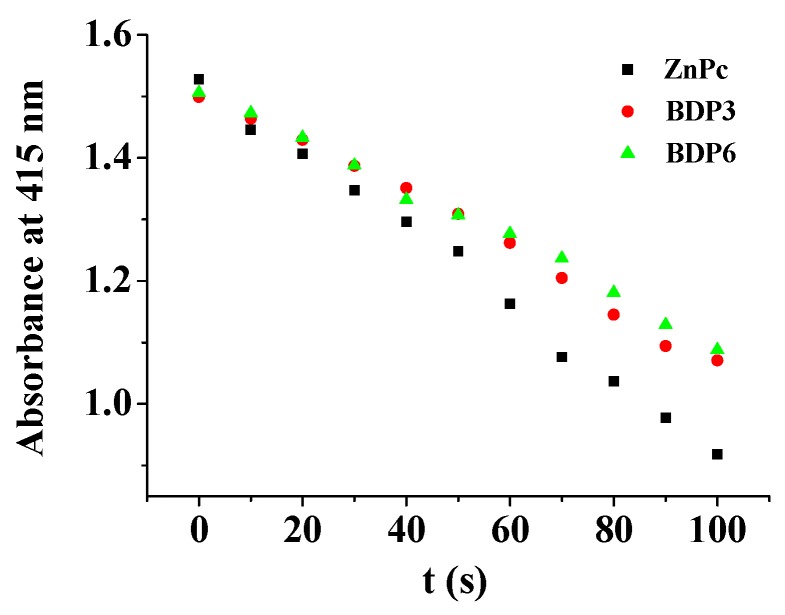
Comparison of the degradation rates of 1,3-diphenylisobenzofuran (DPBF) in DMF as monitored spectroscopically at 415 nm, using **BDP3** and **BDP6** as the photosensitizers and unsubstituted zinc(II) phthalocyanine (ZnPc) as the standard.

The electronic absorption as well as basic photo-physical and photo-chemical properties of these two trifluoromethyl BODIPY derivatives **BDP3** and **BDP6** was investigated in *N*,*N*-dimethylformamide (DMF) and the data are summarized in Table 1. As shown in Figure 1, both compounds give typical absorption spectra of non-aggregated BODIPY, showing an intense and sharp Q-band at 638 (or 640) nm, which strictly follows the Lambert–Beer’s law. Upon excitation at 610 nm, **BDP3** and **BDP6** show a narrow fluorescence emission at 662 and 661 nm, respectively. The fluorescence quantum yields (Φ_F_) were determined to be 017 and 0.16 by using unsubstituted zinc(II) phthalocyanine (ZnPc) (Φ_F_ = 0.28) as the standard compound (Table 1) [18]. To evaluate the photosensitizing potential, the singlet oxygen quantum yields (Φ_Δ_) of these two BODIPY derivatives were also determined in DMF by a steady-state method using 1,3-diphenylisobenzofuran (DPBF) as the singlet oxygen scavenger and ZnPc as the standard. The concentration of the quencher was monitored spectroscopically at 415 nm along with time, from which the values of Φ_Δ_ could be determined by the method described previously [19]. The data are also summarized in Table 1. Figure 2 compares the rates of decay of DPBF using the two BODIPYs and ZnPc as the photosensitizers. It can be seen that the two trifluoromethyl BODIPY derivatives are efficient singlet oxygen generators (Φ_Δ_ = 0.36 and 0.32) (Table 1). These results show that **BDP3** and **BDP6** have similar electronic absorption, fluorescence emission and singlet oxygen generation efficiency.

### 2.3. In Vitro Studies

#### 2.3.1. Photocytotoxicity Studies

**Figure 3:**
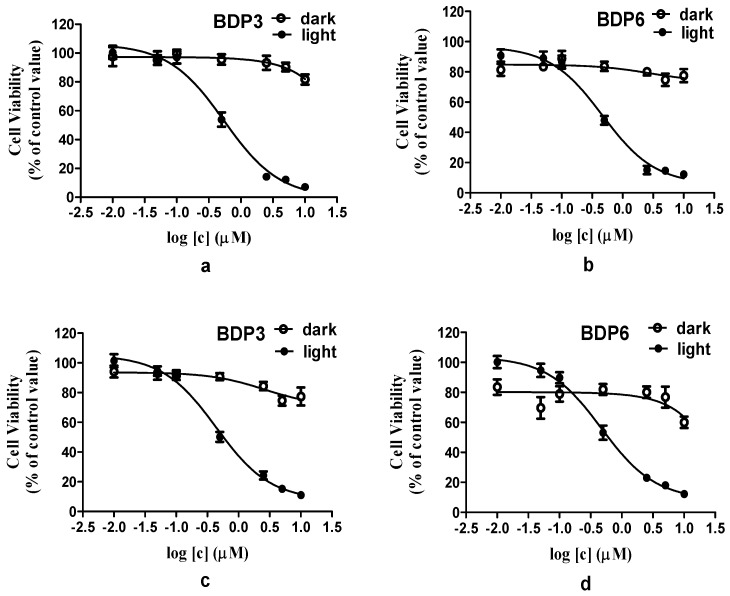
Effects of **BDP3** and **BDP6** on HepG2 (**a**,**b**) and HeLa (**c**,**d**) cells in dark (○) and in light (●) (λ = 660 nm, 1.5 J/cm^2^). Data are expressed as Mean ± SEM of three independent experiments; six replicates were used for each concentration in each experiment.

BODIPY derivatives usually have a low solubility and tend to aggregate in biological media. Cremophor EL is a well-known surfactant, it was often used to promote the dissolution of hydrophobic photosensitizers and reduce their aggregation behavior in biological media [35]. Similarly, in this report, Cremophor EL was added to enhance the solubility and dispersion of BODIPYs in biological media. The in vitro photodynamic activities of photosensitizers **BDP3** and **BDP6** in Cremophor EL emulsions were investigated against two different cell lines (HepG2 and HeLa cancer cells). The assay of methyl thiazolyl tetrazolium (MTT, Gen View Co., Ltd., Tallahassee, FL, USA) was employed to determine the cytotoxicity of **BDP3** and **BDP6** (Figure 3, Supplement Tables S1–S4). As shown in the dose-dependent survival curves (Figure 3), both compounds have the essentially negligible cytotoxicity in the absence of light up to 2 μM, but exhibit a high phototoxicity to photosensitizer-stained HepG2 or HeLa cells upon irradiation with the light dosage of 1.5 J/cm^2^ (λ = 660 nm). The corresponding IC_50_ values, defined as the dye concentrations required to kill 50% of the cells, are summarized in Table 2. For HepG2 cells, the IC_50_ values of **BDP3** and **BDP6** were 0.49 μM and 0.45 μM, respectively. For HeLa cells, the IC_50_ values of these two compounds were 0.42 μM and 0.45 μM. Their in vitro photocytotoxicity exceeds the level attained by Foscan, a clinically used photosensitizing drug (IC_50_ = 0.8 μM at a light dosage of 1.8 J/cm^2^) [36]. The two BODIPYs have superior photodynamic activities and similar highly potency and effects on HepG2 and HeLa cells, indicating that the two BODIPYs may be used as photosensitizer agents for treating a wide range of cancers.

**Figure 4:**
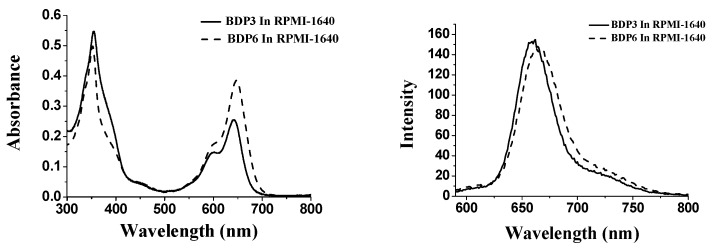
Electronic absorption (**left**) and Fluorescence emission spectra (**right**) of **BDP3** and **BDP6**, formulated with Cremophor EL (0.05%) in the RPMI 1640 culture medium (all at 10 μM).

With the large π-conjugated structure, BODIPY derivatives always tend to aggregate in the aqueous solution, as a result, their photocytotoxicities are significantly reduced or even disappeared [23]. To account for the in vitro photodynamic activities, the aggregation behavior of photosensitizers **BDP3** and **BDP6**, formulated with Cremophor EL in the RPMI 1640 culture medium, was examined by the electronic absorption and fluorescence spectroscopic methods (Figure 4). It can been seen that the Q-bands of all these compounds remain sharp and intense, meanwhile strong fluorescence emission peaks are observed at ca. 662 nm upon excitation at 610 nm indicating the two BODIPYs are not significantly aggregated under these conditions, which seems to accord with their high photodynamic activities.

#### 2.3.2. Measurements of Intracellular ROS

**Figure 5:**
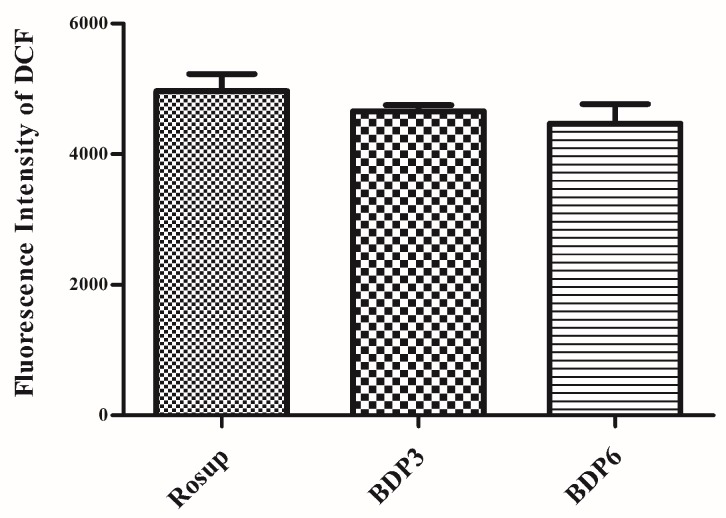
Intracellular 2′,7′-dichlorofluorescein (DCF) fluorescence of HepG2 cells after incubation with **BDP3** (0.5 μM), **BDP6** (0.5 μM) for 24 h at 37 °C, and then under light irradiation for 20 min with a LED lamp of light dosage of 1.5 J/cm^2^ (λ = 660 nm). The culture media with Rosup (50 μg/mL) were set as the positive controls. Values are Mean ± SEM of three independent experiments; six replicates were used for each experiment.

The irradiation of the photosensitizers leads to the production of ROS, which is thought to be the main mediator of cellular death induced by PDT and plays an important role in apoptosis [7]. ROS can mediate cellular effects such as lipid peroxidation and vascular effects, thus resulting in direct or indirect cytotoxicity effects on the treated cells. Here, the intracellular ROS generation efficiency of both BODIPYs against HepG2 cells was also examined with 2′,7′-dichlorodihydrofluorescein diacetate (DCFH-DA) as the indicator [37]. As previously reported, DCFH-DA is non-fluorescent and its oxidized product 2′,7′-dichlorofluorescein (DCF) by ROS can emit green fluorescence. The treatment of DCFH-DA with **BDP3**, **BDP6** and Rosup (positive control) induced a strong fluorescence (Figure 5, Table 3). These findings suggest that the two BODIPYs have the high ROS generation ability and show no obvious difference. The results are consistent with the MTT assay results. As a negative control, the culture medium without agent has no effect on the ROS levels. 

#### 2.3.3. Subcellular Localization

**Figure 6:**
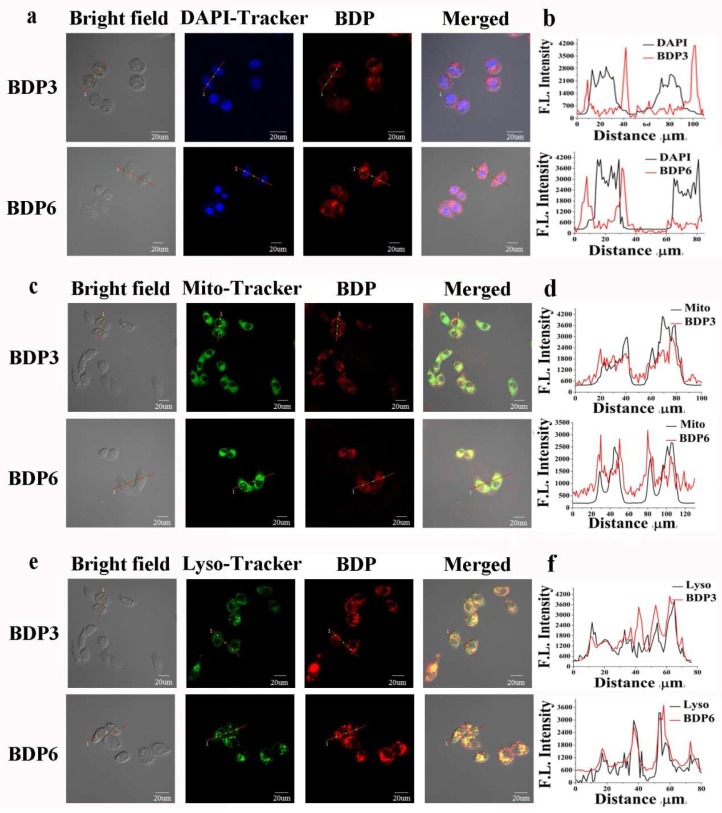
Visualization of the intracellular fluorescence of HepG2 cells for DAPI (in blue, **a**), Mito-Tracker Green (in green, **c**), Lyso-Tracker (in green, **e**), and **BDP3** or **BDP6** (in red, 2 μM). Fluorescence intensity profiles of DAPI (**b**), Mito-Tracker Green (**d**), Lyso-Tracker (**f**), and **BDP3** or **BDP6** (2 μM) traced along the red line in (**a**,**c**,**e**).

Cellular uptake and subcellular localization of **BDP3** and **BDP6** in the HepG2 and HeLa cells were also carried out by an Olympus FV1000 Confocal Laser Scanning Microscope (Olympus Instrument Co., Ltd., Shinjuku-ku, Tokyo, Japan). The cells were first incubated with **BDP3** or **BDP6** in the culture medium for 24 h and then stained with 4,6-diamidino-2-phenylindole (DAPI) (incubated for 10 min), Mito-Tracker Green (incubated for 30 min) or Lyso-Tracker DND-26 (incubated for 1 h), which were respectively specific dyes for nucleus, lysosomes, and mitochondria. After incubation with these two compounds for 24 h and upon excitation at 633 nm, the HepG2 and HeLa cells showed high intracellular fluorescence (Figure 6 and Figure S1), indicating that there was a substantial uptake of the photosensitizers. As shown in Figure 6c,e, against HepG2 cells, both the fluorescence caused by the Mito-Tracker Green (excited at 488 nm, monitored at 510–570 nm) or Lyso-Tracker DND-26 (excited at 488 nm, monitored at 510–570 nm) can superimpose on the fluorescence caused by photosensitizers **BDP3** and **BDP6** (excited at 633 nm, monitored at 650–750 nm). Furthermore, the similar fluorescence intensity profiles (Figure 6d,f) traced along the red line also confirm that these two BODIPYs can target mitochondria and lysosomes of cells well. The fluorescence images of **BDP3** or **BDP6** cannot superimpose on that of DAPI (excited at 405 nm, monitored at 425–475 nm) (Figure 6a,b), indicating that both BODIPYs are not localized in the nucleus. The same results were observed in the HeLa cells (Figure S1).

## 3. Materials and Instruments

Dichloromethane and toluene were distilled from calcium hydride and sodium, respectively. Chromatographic purifications were performed on silica gel (Qingdao Ocean, Qingdao, Shandong, China, 200–300 mesh) columns with the indicated eluents. RPMI 1640 cell culture medium (Gibco, Grand Island, NY, USA) was purchased from Ding Biotechnology Co., Ltd., Haimen, China. HepG2 human hepatocarcinoma and HeLa human cervical carcinoma cells were obtained from the cell bank of Shanghai Institutes for Biological Sciences. The methyl thiazolyl tetrazolium (MTT, Gen View Co., Ltd., Tallahassee, FL, USA) assay was performed on a 96-well sterile cell culture plate (NEST Biotechnology Co., Ltd., Wuxi, Jiangsu, China). All other solvents and reagents were of analytical grade and used as received. 

^1^H-NMR spectra were recorded on AVANE III 400 (^1^H, 400 MHz) instrument (Bruker, Karlstuhe, Germany) in CDCl_3_. Chemical shifts were expressed in ppm relative to TMS (0 ppm). High-resolution mass spectra (HRMS) analysis was carried out on an Agilent 6520 ACURATE-Mass Q-TOF Mass Spectrometer (Agilent Technologies, Santa Clara, CA, USA). Electronic absorption spectra were measured on a Beijing Puxi Tu-1901 Spectrometer (Beijing PuXi Spectrum Optical Instrument Co., Ltd., Beijing, China) and fluorescence spectra were obtained on a VARIAN Carye Elipse Fluorescence spectrometer (Agilent Technologies, Santa Clara, CA, USA). Subcellular localization was carried out on an Olympus FV1000 Confocal Laser Scanning Microscope (Olympus Instrument Co., Ltd., Shinjuku-ku, and Tokyo, Japan)

### 3.1. Synthesis

#### 3.1.1. Synthesis of **1a**

To the solution of 4-trifluoromethylbenzaldehyde (1.50 g, 9.30 mmol) and 2,4-dimethylpyrrole (1.82 g, 19.08 mmol) in anhydrous dichloromethane (80 mL) was added one drop of trifluoroacetic acid. The mixture was stirred overnight at ambient temperature and then the solution of 2,3-dichloro-5,6-dicyano-p-benzoquinone (2.12 g, 9.35 mmol) in anhydrous dichloromethane (150 mL) was added. The resulting mixture was stirred continuously for another 4 h. After the addition of triethylamine (18 mL, 0.13 mol), BF_3_·Et_2_O (18 mL, 0.15 mol) was dropwise added into the mixture, which was cooled in an ice-water bath. The mixture was stirred overnight at ambient temperature and then filtered through a celite pad to remove black solid impurities. The filtrate was then washed with saturated NaHCO_3_ aqueous solution (200 mL × 2), followed by saturated NaCl aqueous solution (200 mL × 2). The organic fraction was dried over anhydrous Na_2_SO_4_ and then concentrated to dryness under vacuum. The crude product was purified by silica gel column chromatography using CH_2_Cl_2_ /petroleum ether (1:2, *v*/*v*) as the eluent to give **1a** as the orange-yellow solid (1.10 g, 30%). ^1^H-NMR (400 MHz, CDCl_3_): δ 7.78 (d, *J* = 8.0 Hz, 2H, ArH), 7.46 (d, *J* = 7.6 Hz, 2H, ArH), 6.01 (s, 2H, pyrrole-H), 2.56 (s, 6H, CH_3_), 1.35 (s, 6H, CH_3_) ppm. HRMS (ESI): *m*/*z* calcd for C_20_H_1__9_BF_5_N_2_ [M + H]^+^: 393.1556, found 393.1550 [38].

#### 3.1.2. Synthesis of **1b**

A similar procedure was employed to prepare compound **1b** with a yield of 23% by replacing 4-(trifluoromethyl)benzaldehyde wth 3,5-bis(trifluoromethyl)benzaldehyde. ^1^H-NMR (400 MHz, CDCl_3_): δ 8.03 (s, 1H, ArH), 7.84 (s, 2H, ArH), 6.03 (s, 2H, pyrrole-H), 2.57 (s, 6H, CH_3_), 1.32 (s, 6H, CH_3_) ppm. HRMS (ESI): *m*/*z* calcd for C_21_H_1__8_BF_8_N_2_ [M + H]^+^: 461.1430, found 461.1425.

#### 3.1.3. Synthesis of **2a**

The mixture of compound **1a** (0.15 g, 0.39 mmol), I_2_ (0.39 g, 1.56 mol) and HIO_4_ (0.23 g, 1.25 mmol) in absolute ethanol (200 mL) was stirred under an atmosphere of nitrogen for 6 h at 60 °C. The mixture was concentrated under reduced pressure after the reaction was completed, as monitored by thin-layer chromatography (TLC). Then the residue was purified by silica gel column chromatography using CH_2_Cl_2_/petroleum ether (1:2, *v*/*v*) as the eluent to give **2a** as a red solid (0.31 g, 83%). ^1^H-NMR (400 MHz, CDCl_3_): δ 7.82 (d, *J* = 7.6 Hz, 2H, ArH), 7.44 (d, *J* = 8.0 Hz, 2H, ArH), 2.66 (s, 6H, CH_3_), 1.36 (s, 6H, CH_3_) ppm. HRMS (ESI): *m*/*z* calcd for C_20_H_1__6_BF_5_I_2_N_2_ [M]^+^: 643.9411, found 643.9409.

#### 3.1.4. Synthesis of **2b**

According to the above procedure, **1b** (0.17 g, 0.37 mol) was treated with I_2_ (0.24 g, 0.93 mol) and HIO_4_ (0.13 g, 0.74 mol) in absolute ethanol to afford **2b** (0.20 g, 75%). ^1^H-NMR (400 MHz, CDCl_3_): δ 8.09 (s, 1H, ArH), 7.82 (s, 2H, ArH), 2.66 (s, 6H, CH_3_), 1.34 (s, 6H, CH_3_) ppm. HRMS (ESI): *m*/*z* calcd for C_21_H_1__5_BF_8_I_2_N_2_ [M]^+^: 711.9290, found 711.9303.

#### 3.1.5. Synthesis of **BDP3**

A mixture of compound 2a (0.15 g, 0.24 mmol), 4-trifluoromethylbenzaldehyde (0.41 g, 16 mmol), piperidine (0.90 mL), glacial acetic acid (0.75 mL) and a small amount of Mg(ClO_4_)_2_ in anhydrous toluene (50 mL) was refluxed for 2 h. Water generated in the reaction was removed azeotropically with a Dean–Stark apparatus. The volatiles was removed under reduced pressure. The resulting residue was dissolved in dichloromethane and washed with water (200 mL × 2). The organic fraction was collected and purified by silica gel column chromatography using CH_2_Cl_2_/ petroleum ether (2:1, *v*/*v*) as the eluent. **BDP3** was obtained as a green solid (0.0052 g, 21%). ^1^H-NMR (400 MHz, CDCl_3_): δ 8.17 (d, *J* = 16.4 Hz, 2H, CH=CH), 7.86 (d, *J* = 8.0 Hz, 2H, ArH), 7.75 (d, *J* = 8.4 Hz, 4H, ArH), 7.74 (d, *J* = 16.0 Hz, 2H, CH=CH), 7.68 (d, *J* = 8.0 Hz, 4H, ArH), 7.50 (d, *J* = 8.0 Hz, 2H, ArH), 1.46 (s, 6H, CH_3_) ppm. HRMS (ESI): *m*/*z* calcd for C_36_H_22_BF_11_I_2_N_2_ [M]^+^: 955.9790, found 955.9812.

#### 3.1.6. Synthesis of **BDP6**

According to the above procedure, **2b** (0.10 g, 0.14 mmol) was treated with 3,5-bis(trifluoromethyl)benzaldehyde (0.34 g, 20 mmol), piperidine (0.90 mL), and acetic acid (0.75 mL) to give **BDP6** (0.037 g, 23%) as a green solid. ^1^H-NMR (400 MHz, CDCl_3_): δ 8.22 (d, *J* = 16.8 Hz, 2H, CH=CH), 8.15 (s, 1H, ArH), 8.03 (s, 4H, ArH), 7.90 (s, 2H, ArH), 7.86 (s, 2H, ArH), 7.78 (d, *J* = 16.8 Hz, 2H, CH=CH), 1.45 (s, 6H, CH_3_) ppm. HRMS (ESI): *m*/*z* calcd for C_3__9_H_19_BF_20_I_2_N_2_ [M]^+^: 1159.9411, found 1159.9422.

### 3.2. Photo-Physical and Photo-Chemical Studies

#### 3.2.1. Absorption and Fluorescence Studies

UV-Vis and steady-state fluorescence spectra were obtained on a Beijing PuXi Tu-1901 spectrometer and a VARIAN Carye Elipse fluorescence spectrometer, respectively. The samples (**BDP3** and **BDP6**) were dissolved in DMF in a quartz colorimetric utensil. The molar extinction coefficient was determined according to Lambert–Beer’s law. Fluorescence emission spectra were recorded from 600 nm to 900 nm under the excitation at 610 nm. Both the excitation and emission slits were set to 5 nm. The fluorescence quantum yields were determined as:

Φ_F_= (F/F_std_)(A_std_/A) Φ_F(std)_ (1)

where F and F_std_ are the measured fluorescence area (λ_ex_ = 610 nm, area under the emission peak) of the BODIPYs and standard; A and A_std_ are the absorbances at the excitation wavelength (610 nm) of the BODIPYs and standard. Here, the unsubstituted zinc(II) phthalocyanine (ZnPc) in DMF was used as the standard [Φ_F_ = 0.28]. To minimize re-absorption of the radiation by the ground-state species, the emission spectra were obtained by controlling the absorbances of BODIPYs and ZnPc at 610 nm were within the range from 0.03 to 0.05.

#### 3.2.2. Detection of Singlet Oxygen (^1^O_2_) Generation Efficiency

Singlet oxygen quantum yields (Φ_Δ_) were indirectly measured according to our previous work [19]. In brief, 1,3-diphenylisobenzofuran (DPBF) was used as the scavenger and ZnPc was selected as the standard (Φ_Δ_ = 0.56 in DMF). A mixture of DPBF (1 mM) (absorbance at 415 nm ≈ 1.6) and photosensitizer (absorbance of Q-band ≈ 1.0) in DMF (3 mL) was illuminated with laser (670 nm, 20 mW). The decay of the DPBF absorption at 415 nm was monitored. The singlet oxygen quantum yields of photosensitizers were calculated as:

Φ_Δ_= Φ_Δ__std_(k/k_std_)(I_std_/I) (2)

where Φ_Δ__std_ is the quantum yield of singlet oxygen formulation for ZnPc; k and k_std_ are respectively the DPBF photobleaching rates in the presence of **BDP3** (or **BDP6**) and ZnPc; I_std_ and I are the rates of light absorbed by **BDP3** (or **BDP6**) and ZnPc.

### 3.3. In Vitro Studies

#### 3.3.1. Cell Culture and Conditions

HepG2 human hepatocarcinoma and HeLa human cervical carcinoma cells were maintained in RPMI 1640 medium (Gibco) supplemented with Fetal Calf Serum (10%, *v*/*v*), penicillin–streptomycin (0.1%, *v*/*v*). These two cell lines were both cultured at 37 °C in a humidified atmosphere with 5% CO_2_. Approximately 7 × 10^4^ (for HepG2 and HeLa) cells per well in these media were inoculated in 96-multiwell plates and incubated overnight at 37 °C in a humidified 5% CO_2_ atmosphere.

#### 3.3.2. Photocytotoxicity Studies

Photosensitizers (**BDP3** and **BDP6**) were first dissolved in dimethyl sulfoxide (DMSO) containing 5% Cremophor EL to give 1.0 mM solutions, which were diluted to 10 μM with the ultrapure water. The solutions were filtered with a 0.2 μm filter, then diluted with the culture medium to appropriate concentrations (0.001, 0.005, 0.01, 0.1, 0.5, 1.0, and 2.0 μM). The final contents of DMSO and Cremophor EL in the drug solutions were less than 0.2% and 0.01%, respectively. The culture media containing 0.2% (*v*/*v*) DMSO and 0.01% (*v*/*v*) Cremophor EL as the controls are non-cytotoxicity in the presence and absence of light proved by our experiments (Figures S2 and S3). Approximately 7 × 10^3^ cells (HepG2 and HeLa) per well in these media were inoculated in 96-multiwell plates and incubated overnight at 37 °C in a humidified atmosphere with 5% CO_2_. The cells, after being rinsed with PBS, were incubated with 100 μL of these photosensitizer solutions at 37 °C under 5% CO_2_. After 24 h incubation, the medium containing drugs was replaced by fresh medium and then the cells were illuminated at a light dosage of 1.5 J/cm^2^ with a red LED lamp (λ = 660 nm) for 20 min. 

Cell viability was determined by the colorimetric MTT assay [39]. After illumination, the cells were incubated for another 24 h and then a MTT solution in PBS (10 μL, 4 mg/mL) was added to each well. The plates were incubated again for 4 h. The medium was removed carefully and DMSO (100 μL) was added to each well. The absorbance of the solution at 570 nm in each well was taken by a microplate reader. The average absorbance of the blank wells, which did not contain the cells, was subtracted from the readings of the other wells. The cell viability was then determined by the following equation: % viability = [∑(A_i_/A_control_ × 100)]/n, where A_i_ is the absorbance of the ith data (i = 1, 2, …, n), A_control_ is the average absorbance of the control wells in which the BODIPY was absent, and n (= 6) is the number of the data points. Six replicates were used for each concentration in each experiment and each experiment was repeated three times. The procedures for investigation of dark-cytotoxicity were almost the same except that there was no irradiation. The dose-dependent survival curves were prepared with GraphPad Prism 5.0 Software (GraphPad Software Inc., La Jalla, CA, USA) and IC_50_ was calculated.

#### 3.3.3. Measurements of Intracellular ROS

ROS was measured on the basis of the intracellular peroxide-dependent oxidation reaction of DCFH-DA [37] to form the fluorescent compound DCF. HepG2 cells were seeded onto a 96-well plate at a density of 5 × 10^3^ cells per well and cultured overnight. Then fresh medium containing drugs (0.5 μM, 0.01% Cremophor EL) was added and the cells were incubated for 24 h in dark. After washing three times with PBS, 40 μL of DCFH-DA (10 μM) was added and the cells were incubated for another 30 min. The medium was removed and the cells were washed three times with PBS, followed by illumination at a light dosage of 1.5 J/cm^2^ with a red LED lamp (λ = 660 nm) for 20 min. After illumination, the medium was gently removed and then a solution of sodium dodecyl sulfate (SDS, Sigma; 1% by weight, 120 μL) was then added to each well. The plate was agitated for 20 min on a constant temperature shaker. Then the DCF fluorescence was measured by a Bio-Tek microplate reader (Corning Incorporated., New York, NY, USA) (excitation/emission: 488/525 nm). The culture medium containing 50 μg/mL Rosup (a compound mixture, provided by the assay kit) was used as a positive control. The culture medium containing no agent was used as a negative control. Both of them were subjected to irradiation with a light dosage of 1.5 J/cm^2^. Six replicates were used for each experiment and each experiment was repeated three times. 

#### 3.3.4. Subcellular Localization Studies

Approximately 1 × 10^4^ HepG2 or HeLa cells in logarithmic growth phase were added on a cell culture dish and incubated overnight. Then the medium was replaced by fresh medium containing drugs (2 μM) and the cells were incubated for 24 h again. After incubation, the cells were rinsed with PBS three times and incubated with DAPI (Beyutime Institute of Biotechnology Co., Ltd., Haimen, China, 2 μM in culture medium, incubated for 10 min), Mito-Tracker Green (Beyutime Institute of Biotechnology Co., Ltd., Haimen, China, 2 μM in culture medium, incubated for 30 min), or Lyso-Tracker DND-26 (Xiamen Bioluminor Bio-Technology Co., Ltd., Xiamen, China, 2 μM in culture medium, incubated for 1 h). Then the cells were rinsed with PBS three times again and examined with an Olympus FV1000 Confocal Laser Scanning Microscope (Olympus Instrument Co., Ltd., Shinjuku-ku, Tokyo, Japan). Specimens were excited by laser at 405 nm (DAPI), 488 nm (Mito-Tracker Green), 488 nm (Lyso-Tracker DNd-26) or 633 nm (**BDP3** or **BDP6**), and the fluorescence emissions (the first one (blue, 425–475 nm), the second one (green, 510–570 nm), the third one (green, 510–570 nm), and the last one (red, 650–750 nm)) were collected.

## 4. Conclusions

We have successfully synthesized two trifluoromethyl BODIPY derivatives (**BDP3** and **BDP6**) and investigated their photo-physical, photo-chemical, and photo-biological properties. The results show that the two BODIPYs, formulated with Cremophor EL in the RPMI 1640 culture medium, are slightly aggregated and mainly exist in the monomeric form in the biological medium, leading to their high cellular ROS generation efficiency; moreover, the two photosensitizers are specially localized in the mitochondria and lysosomes exhibiting high photocytotoxicity towards HepG2 and HeLa cancer cells with the IC_50_ values of 0.42–0.49 μM. All these results indicate that the trifluoromethyl BODIPYs are promising antitumor agents for photodynamic therapy.

## Figures and Tables

**Table 1:** Photo-physical/photo-chemical data of **BDP3** and **BDP6** in DMF.

Compounds	λ_max_/nm (log ε)	λ_em_/nm ^a^	Φ_F_^b^	Φ_Δ_^c^
**BDP3**	638 (4.84)	662	0.17	0.36
**BDP6**	640 (5.29)	661	0.16	0.32

^a^ Excited at 610 nm. ^b, c^ Using unsubstituted zinc(II) phthalocyanine (ZnPc) in DMF as the standard (Φ_F_ = 0.28, Φ_Δ_ = 0.56); Φ_F_ is the fluorescence quantum yield; Φ_Δ_ is the singlet oxygen quantum yield.

**Table 2:** IC_50_ values of **BDP3** and **BDP6** against HepG2 and HeLa cells.

Compounds	HepG2	HeLa
	IC_50_ (μM) ^a^	
**BDP3**	0.49	0.42
**BDP6**	0.45	0.45

^a^ Defined as the photosensitizer concentration required to kill 50% of the cells.

**Table 3:** Intracellular 2′,7′-dichlorofluorescein (DCF) fluorescence of HepG2 cells after incubation with **BDP3** (0.5 μM), **BDP6** (0.5 μM) for 24 h at 37 °C.

Compounds	Rosup	BDP3	BDP6
Fluorescence Intensity of DCF	4967.72 ± 154.29	4658.25 ± 60.60	4465.25 ± 164.95

Irradiation for 20 min with a LED lamp of light dosage of 1.5 J/cm^2^ (λ = 660 nm). The culture media with Rosup (50 μg/mL) were set as the positive controls. Values are Mean ± SEM of three independent experiments; six replicates were used for each experiment.

## Supplementary Materials

Supplementary materials are available online at http://www.mdpi.com/1420-3049/23/2/458/s1.
