# Supplementary material for: Trifluoromethyl Boron Dipyrromethene Derivatives as Potential Photosensitizers for Photodynamic Therapy
Source: Molecules. 2018 Feb 19;23(2):458. doi: 10.3390/molecules23020458 (PMC6017863; doi:10.3390/molecules23020458)
Supplement: Supplementary file 1 [file molecules-23-00458-s001.pdf]

Electronic Supporting Information

Trifluoromethyl Boron Dipyrromethene Derivatives as Optimal Photosensitizers for Photodynamic Therapy

Jian-Yong Liu[1,*], Peng-Zhen Zhou[1], Jia-Lin Ma[1] and Xiao Jia[1]

[1] State Key Laboratory of Photocatalysis on Energy and Environment & National & Local Joint Biomedical Engineering Research Center on Photodynamic Technologies, College of Chemistry, Fuzhou University, Fuzhou 350108, P. R. China; E-Mails: 18714968018@163.com (P.-Z. Z.); 973907867@qq.com (J.-L. M.) ; jiaxiao@fzu.edu.cn (X. J.)

* Correspondence: liujianyong82@163.com (J.-Y. L.); Tel.: +86-591-22867105 (J.-Y. L.)

Table of Contents

**Figure S1.**	The subcellular localization of **BDP3** or **BDP 6** in HeLa cells
**Figure S2.**	Effects of culture media without BODIPY as the controls on HepG2 cells
**Figure S3**	Effects of culture media without BODIPY as the controls on HeLa cells
**Figure S4.**	^{1}H NMR spectrum of compound **1a** in $CDCl_3$
**Figure S5.**	HRMS spectrum of compound **1a**
**Figure S6.**	^{1}H NMR spectrum of compound **1b** in $CDCl_3$
**Figure S7.**	HRMS spectrum of compound **1b**
**Figure S8.**	^{1}H NMR spectrum of compound **2a** in $CDCl_3$
**Figure S9.**	HRMS spectrum of compound **2a**
**Figure S10.**	^{1}H NMR spectrum of compound **2b** in $CDCl_3$
**Figure S11.**	HRMS spectrum of compound **2b**
**Figure S12.**	^{1}H NMR spectrum of **BDP3** in $CDCl_3$
**Figure S13.**	HRMS spectrum of **BDP3**
**Figure S14.**	^{1}H NMR spectrum of **BDP6** in $CDCl_3$
**Figure S15.**	HRMS spectrum of **BDP6**
**Table S1.**	The cell viability in light (in dark) of **BDP3** against HepG2 cells
**Table S2.**	The cell viability in light (in dark) of **BDP6** against HepG2 cells
**Table S3.**	The cell viability in light (in dark) of **BDP6** against HeLa cells
**Table S4.**	The cell viability in light (in dark) of **BDP6** against HeLa cells

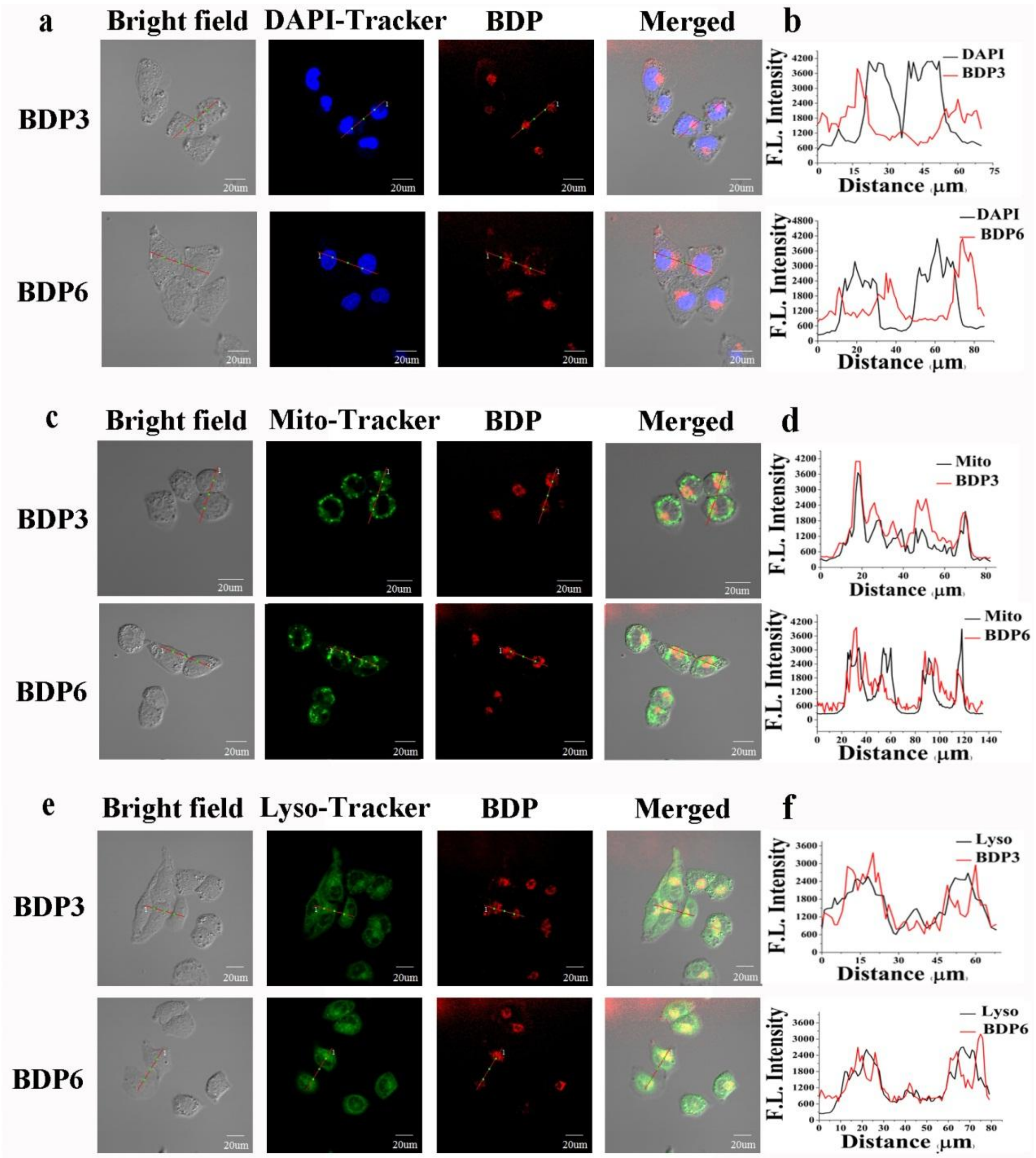

Figure S1. Visualization of the intracellular fluorescence of HeLa cells for DAPI (in blue, a), Mito-Tracker Green (in green, c), Lyso-Tracker (in green, e) and **BDP3** or **BDP6** (in red, 2 μM). Fluorescence intensity profiles of DAPI (b), Mito-Tracker Green (d), Lyso-Tracker (f) and **BDP3** or **BDP6** (2 μM) traced along the red line in (a, c, e).

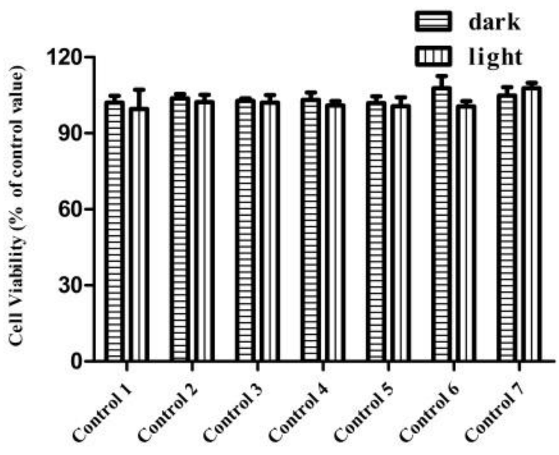

Figure S2. Effects of culture media without BODIPY as the controls on HepG2 cells in dark (cross stripe) and in light (vertical stripe) (λ= 660 nm, 1.5 J/cm²). Data are expressed as Mean ± SEM of three independent experiments; each was performed in six replicates.

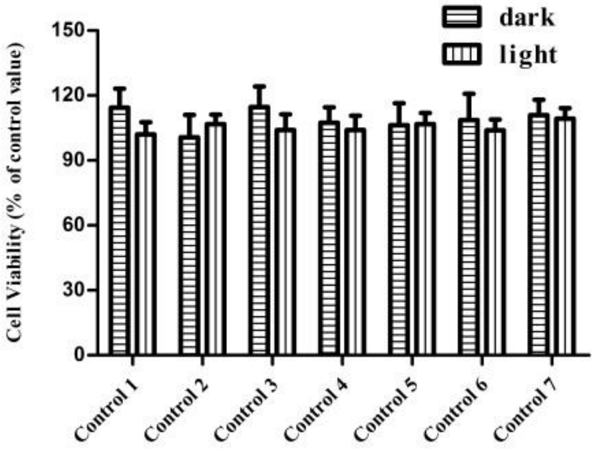

Figure S3. Effects of culture media without BODIPY as the controls on HeLa cells in dark (cross stripe) and in light (vertical stripe) (λ= 660 nm, 1.5 J/cm²). Data are expressed as Mean ± SEM of three independent experiments; each was performed in six replicates.

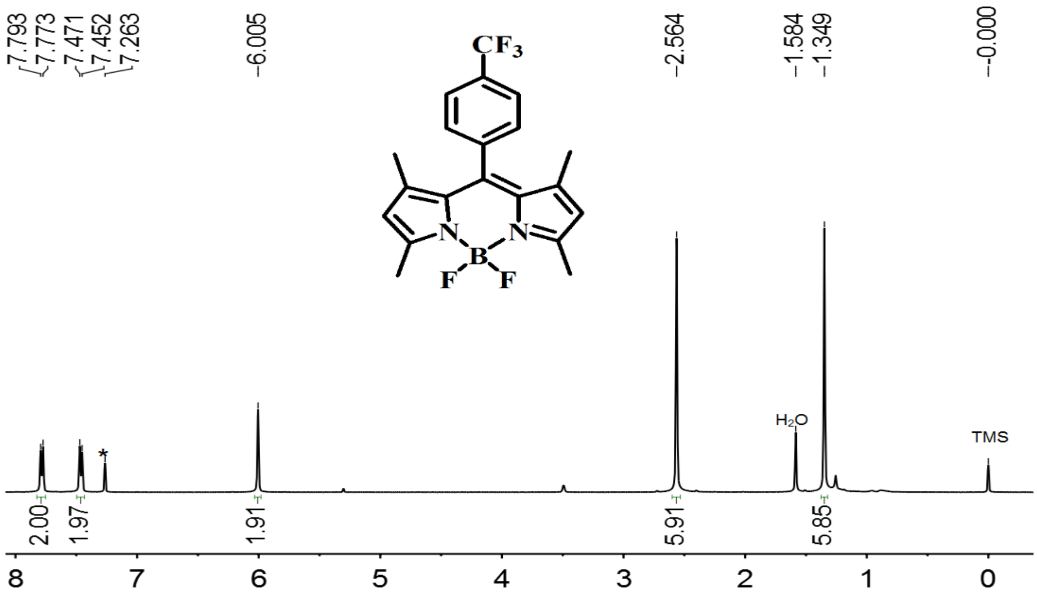

Figure S4. ^1H NMR spectrum of compound **1a** in $CDCl_3$

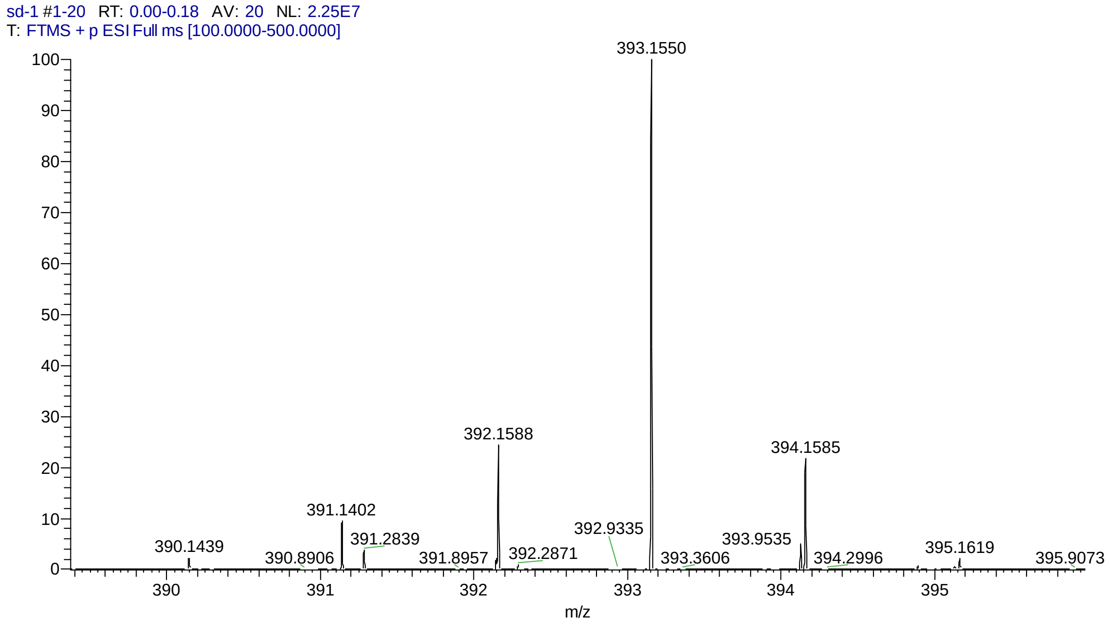

Figure S5. HRMS spectrum of compound **1a**

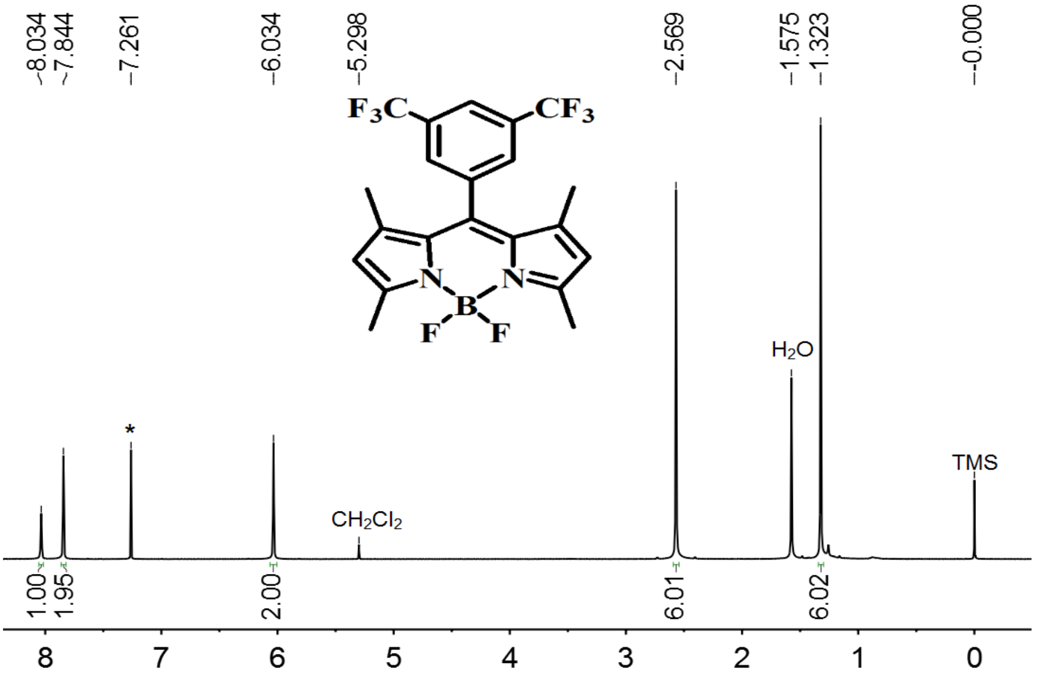

Figure S6. ^{1}H NMR spectrum of compound **1b** in $CDCl_3$

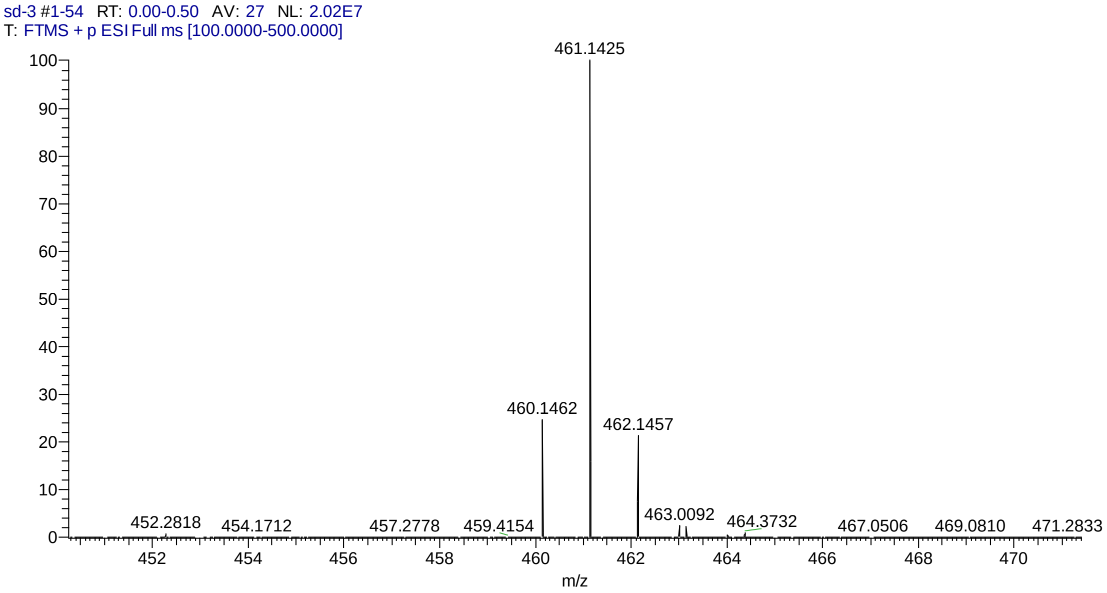

Figure S7. HRMS spectrum of compound **1b**

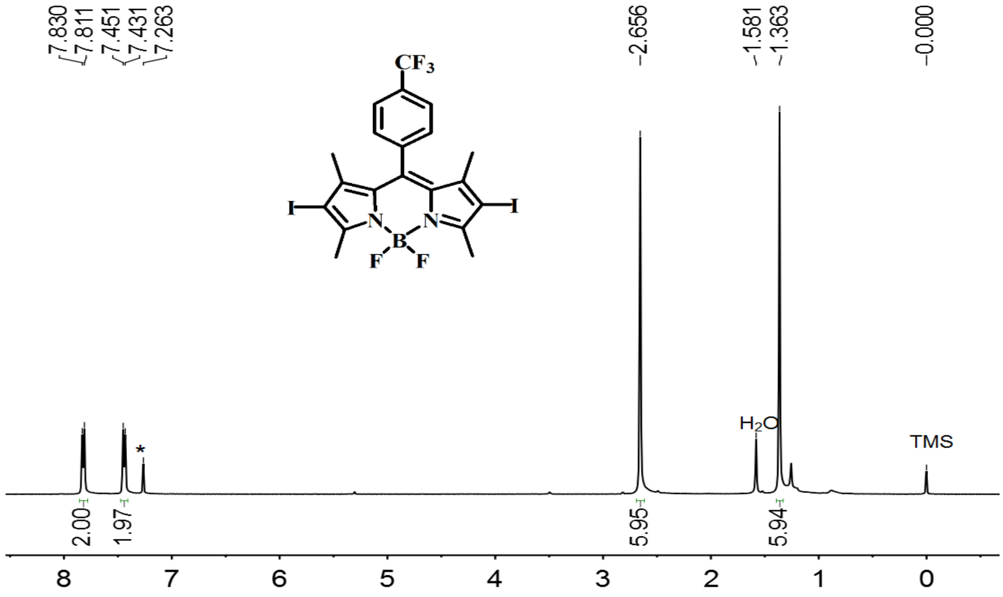

Figure S8. ^{1}H NMR spectrum of compound **2a** in $CDCl_3$

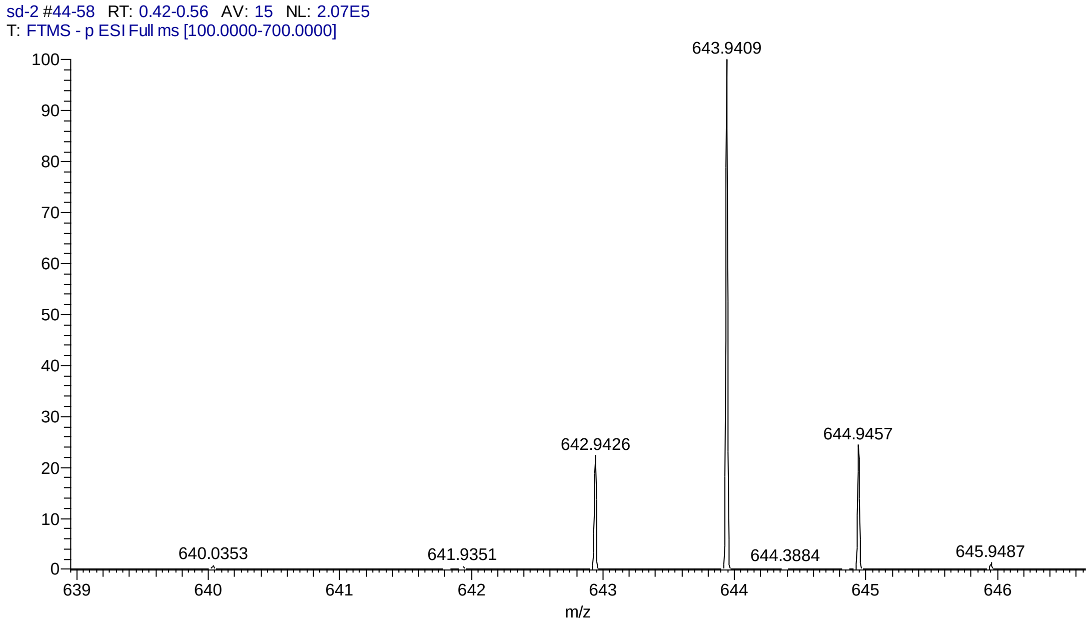

Figure S9. HRMS spectrum of compound **2a**

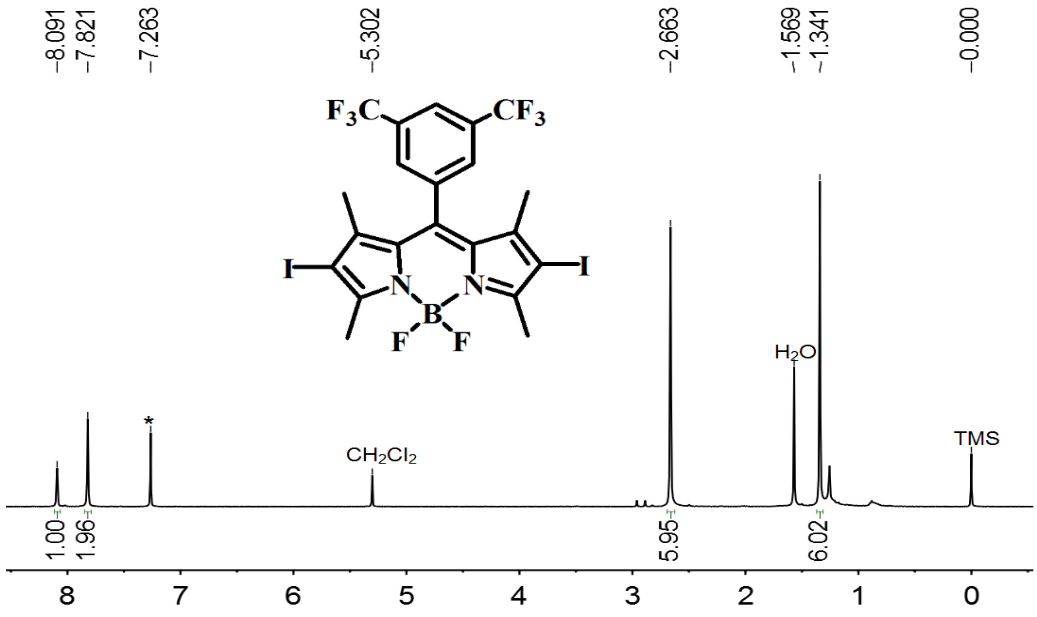

Figure S10. ^{1}H NMR spectrum of compound **2b** in $CDCl_3$

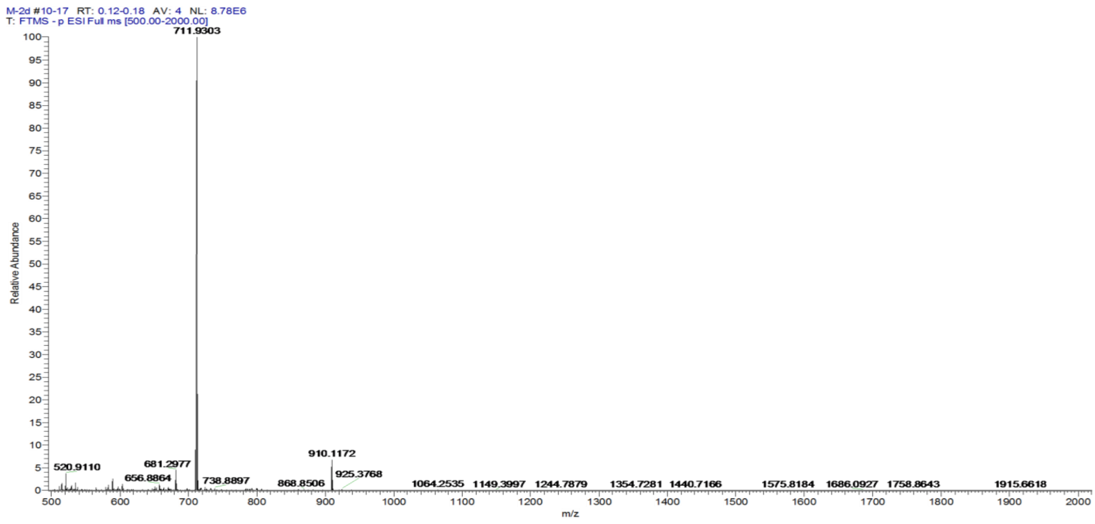

Figure S11. HRMS spectrum of compound **2b**

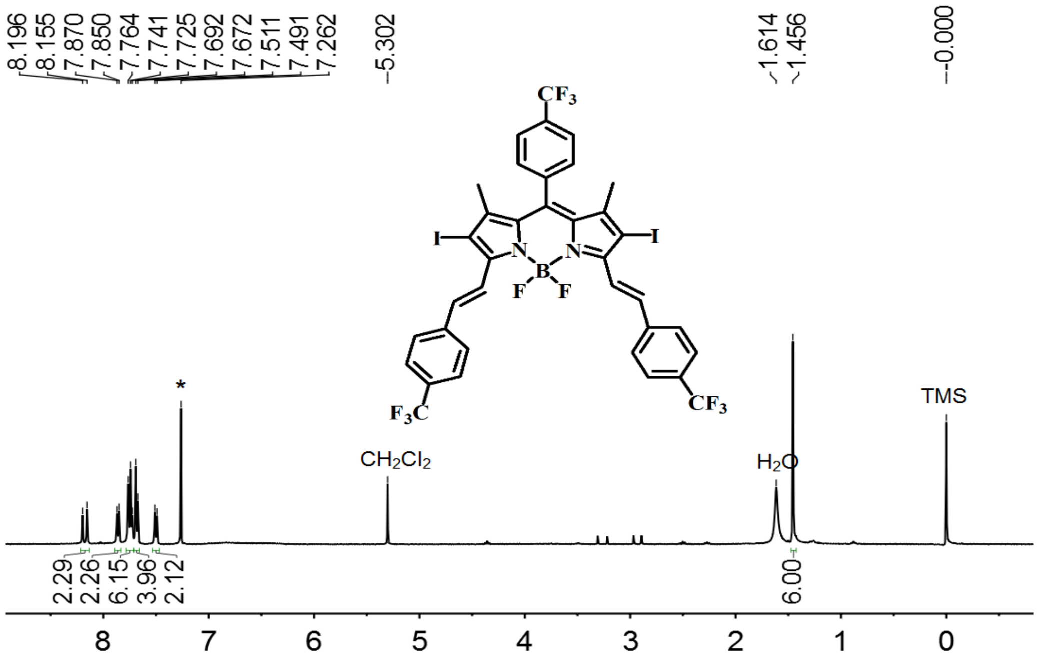

Figure S12. ^{1}H NMR spectrum of **BDP3** in $CDCl_3$

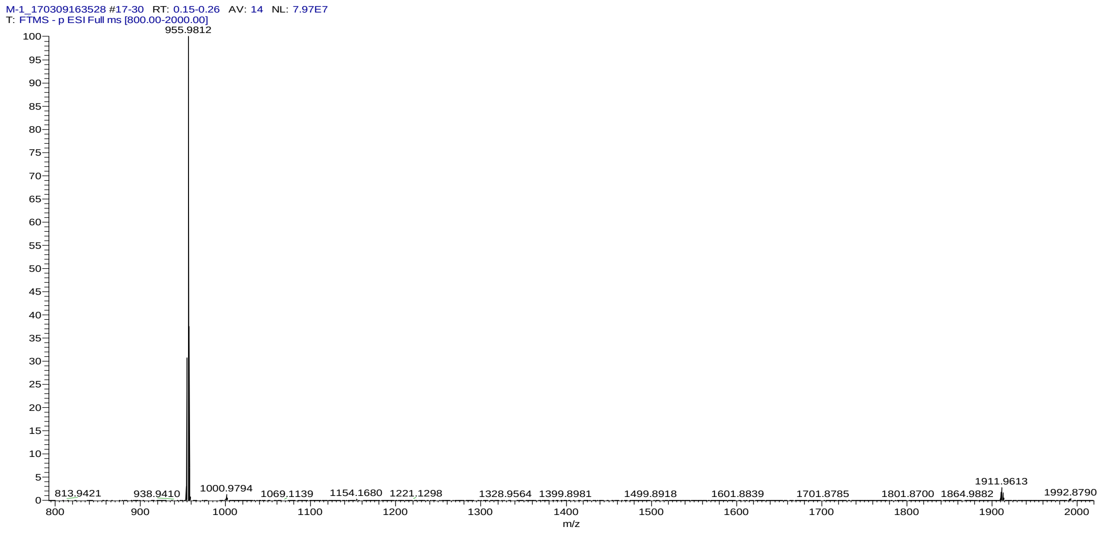

Figure S13. HRMS spectrum of **BDP3**

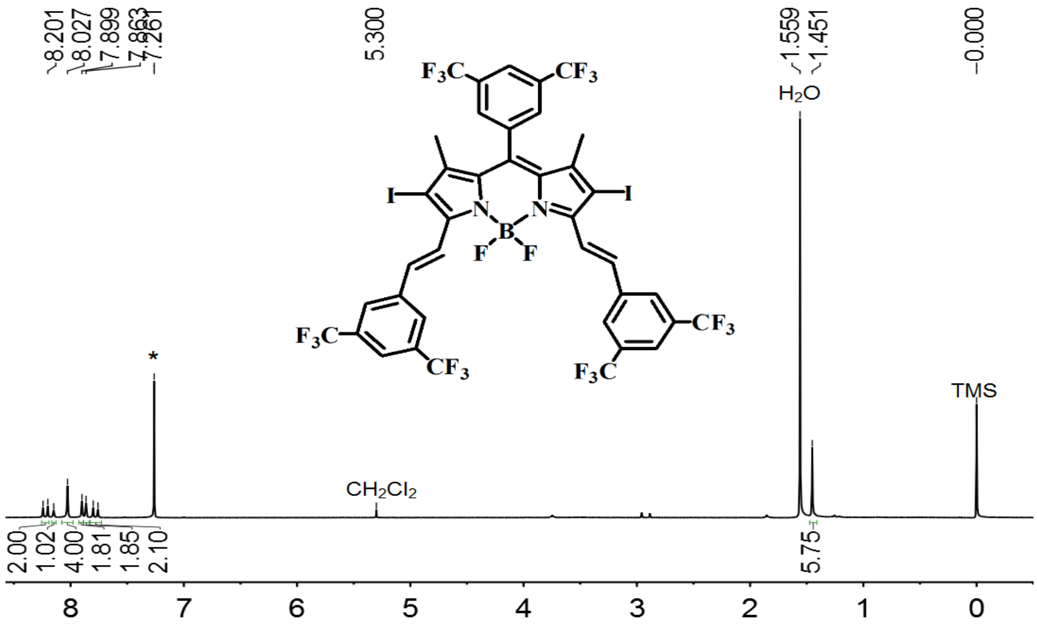

Figure S14. ^{1}H NMR spectrum of **BDP6** in $CDCl_3$

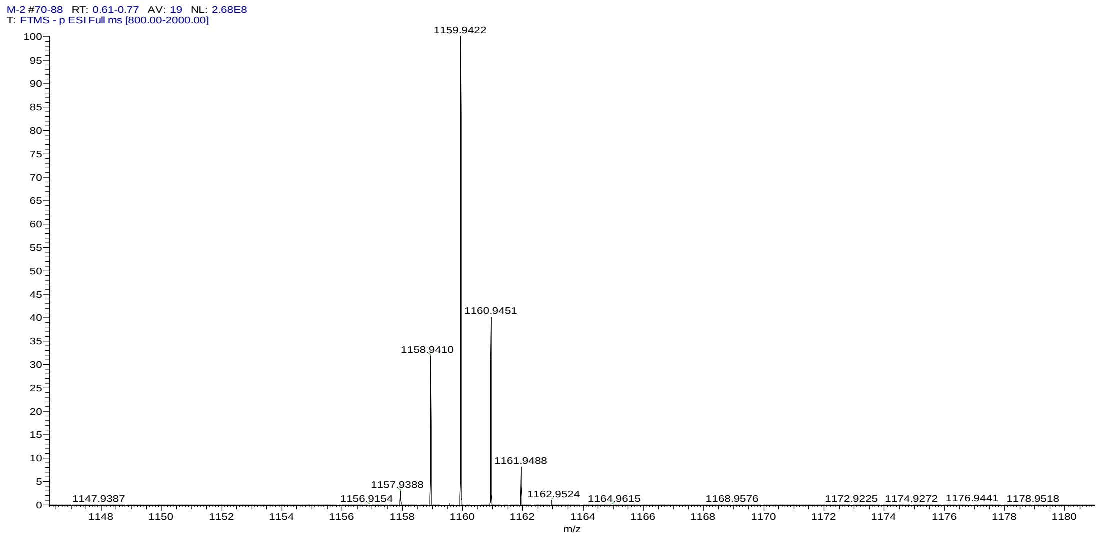

Figure S15. HRMS spectrum of **BDP6**

Table S1. The cell viability in light (in dark) of **BDP3** against HepG2 cells

Concentration /μM	Cell viability in light (%) [a]	Cell viability in dark (%)
10.0	7.156 ± 0.347	81.738 ± 2.922
5.0	12.285 ± 0.739	90.317 ± 2.495
2.5	14.271 ± 0.827	93.223 ± 4.328
0.5	53.888 ± 4.023	95.578 ± 3.036
0.1	97.057 ± 3.578	99.723 ± 1.661
0.05	96.568 ± 3.862	95.141 ± 2.873
0.01	100.488 ± 3.775	97.554 ± 4.389

[a] Irradiation at a light dosage of 1.5 J/cm^2 with LED lamp (λ=660 nm) for 20 min. Data are expressed as Mean ± SEM of three independent experiments; six replicates were used for each concentration in each experiment.

Table S2. The cell viability in light (in dark) of **BDP6** against HepG2 cells

Concentration /μM	Cell viability in light (%) [a]	Cell viability in dark (%)
10.0	12.285 ± 0.739	77.504 ± 3.874
5.0	14.602 ± 1.463	74.739 ± 2.861
2.5	15.105 ± 1.851	80.007 ± 1.645
0.5	47.973 ± 2.125	83.607 ± 2.355
0.1	86.423 ± 3.493	88.733 ± 3.919
0.05	89.062 ± 3.673	83.488 ± 1.294
0.01	90.777 ± 3.242	81.343 ± 3.179

[a] Irradiation at a light dosage of 1.5 J/cm^2 with LED lamp (λ=660 nm) for 20 min. Data are expressed as Mean ± SEM of three independent experiments; six replicates were used for each concentration in each experiment.

Table S3. The cell viability in light (in dark) of **BDP6** against HeLa cells

**Concentration /μM**	**Cell viability in light (%)** [a]	**Cell viability in dark (%)**
10.0	11.081 ± 0.828	77.375 ± 4.764
5.0	15.238 ± 0.940	74.600 ± 2.830
2.5	24.263 ± 2.114	84.376 ± 1.931
0.5	50.199 ± 2.716	90.742 ± 1.743
0.1	91.764 ± 2.313	91.256 ± 2.136
0.05	94.460 ± 2.599	93.149 ± 3.348
0.01	101.450 ± 3.425	94.166 ± 3.151

[a] Irradiation at a light dosage of 1.5 J/cm^2 with LED lamp (λ=660 nm) for 20 min. Data are expressed as Mean ± SEM of three independent experiments; six replicates were used for each concentration in each experiment.

Table S4. The cell viability in light (in dark) of **BDP6** against HeLa cells

**Concentration /μM**	**Cell viability in light (%)** [a]	**Cell viability in dark (%)**
10.0	12.221 ± 0.663	60.055 ± 2.926
5.0	18.084 ± 0.601	76.861 ± 5.221
2.5	23.073 ± 0.547	80.136 ± 2.976
0.5	53.118 ± 3.743	81.928 ± 2.421
0.1	89.747 ± 3.046	78.962 ± 4.288
0.05	94.625 ± 3.417	69.663 ± 5.330
0.01	100.212 ± 3.283	83.503 ± 4.263

[a] Irradiation at a light dosage of 1.5 J/cm^2 with LED lamp (λ=660 nm) for 20 min. Data are expressed as Mean ± SEM of three independent experiments; six replicates were used for each concentration in each experiment.
